# Examining polygenic scores for depression, depressive symptoms from childhood to adolescence, and adolescent substance use in a diverse sample: The moderating impact of a family-centered intervention

**DOI:** 10.1017/S0954579425100709

**Published:** 2025-10-14

**Authors:** Kit K. Elam, Daniel Shaw, Erika Westling, Jazmin Brown-Iannuzzi, Kathryn Lemery-Chalfant

**Affiliations:** 1 Department of Applied Health Science, Indiana University Bloomington, Bloomington, IN, USA; 2 Department of Psychology, University of Pittsburgh, Pittsburgh, PA, USA; 3 Oregon Research Institute, Springfield, OR, USA; 4 Department of Psychology, University of Virginia, Charlottesville, VA, USA; 5 Department of Psychology, Arizona State University, Tempe, AZ, USA

**Keywords:** Adolescence, depressive symptoms, polygenic, substance use, trajectories

## Abstract

Research finds genetic predisposition for depression is associated with increases in depression across adolescence and adulthood. In turn, depressive symptoms in adolescence are associated with substance use. However, there has been modest examination of genetic predisposition for depression, growth in depressive symptoms, and substance use from late childhood through adolescence, and mostly in White samples. Also, psychosocial interventions can attenuate associations between genetic predisposition and psychopathology, a genotype by intervention (GxI) effect. We examined associations among polygenic risk for depression, growth in depressive symptoms from age 7 to 16, and substance use at age 16, as well as moderation by a family-based preventive intervention. Participants were African-ancestry (*n* = 154) and European-ancestry (*n* = 219) youth from the Early Steps Multisite Study, half of whom participated in the Family Check-Up intervention. A small polygenic by intervention effect was found on reductions in depressive symptoms for African-ancestry youth, and growth in depressive symptoms was positively associated with substance use at age 16. In sensitivity analyses, a small GxI effect was detected in European-ancestry youth on reductions in depressive symptom slopes from age 10 to 16. These findings highlight how early intervention can buffer genetic effects on depressive symptoms over time.

## Introduction

Depressive symptoms are relatively stable during childhood but normatively increase across late childhood into adolescence (Musliner et al., 2016; Shore et al., 2018). Depressive symptoms in adolescence are concurrently associated with multiple types of substance use (Hussong et al., 2017). Additionally, genetic predisposition for depression is associated with growth in depressive symptoms across childhood, adolescence, and early adulthood (Elam et al., 2024; Kwong et al., 2019; Lussier et al., 2021; Rice, 2009), but very limited research has examined whether growth in depressive symptoms is associated with substance use outcomes in adolescence. Accumulating evidence also indicates that family-based interventions may buffer genetic influences on psychopathology in youth, a genotype by intervention (GxI) effect (Elam et al., 2022; Lemery-Chalfant et al., 2018; Shaw et al., 2019). Moreover, most genetically informed research on depression in youth is in White samples, limiting the generalizability of results. We address these gaps in the current study by examining associations among polygenic predisposition for depression, growth in depressive symptoms from childhood to adolescence, and substance use outcomes in adolescence in European-ancestry and African-ancestry youth. Further, we examine whether the path from genetic predisposition to growth in depressive symptoms and substance use might be attenuated by a family-based preventive intervention, the Family Check-Up (FCU; Dishion et al., 2008).

### Growth in depressive symptoms from childhood to adolescence

Depressive symptoms are relatively low and stable in childhood but typically increase rapidly during the transition from late childhood to early adolescence (Cohen et al., 2018; Maughan et al., 2013; Shore et al., 2018). These patterns are supported by research and a meta-analysis on growth in depressive symptoms across childhood and adolescence (Musliner et al., 2016; Shore et al., 2018). Theoretically, the transition from childhood to adolescence is accompanied by social stressors and biological processes which can both increase risk for depression (Bernaras et al., 2019; Hussong et al., 2017). However, most of the research on growth in depressive symptoms across childhood and adolescence are in White youth or do not differentiate across racial/ethnic groups. Historically, in adults there is evidence of a Black–White depression paradox in which White individuals have higher rates of depression than Black individuals despite Black individuals experiencing greater exposure to social and structural stressors (Cohen et al., 2018; Mezuk et al., 2013; Pamplin II & Bates, 2021). However, past findings in adolescence are mixed, with most research indicating Black and other racial and ethnic minority youth have greater depressive symptoms and greater positive growth in depressive symptoms compared to White individuals (Musliner et al., 2016; Schubert et al., 2017; Shore et al., 2018). Some evidence indicates that individual and familial strengths in Black youth, such as individual coping and family support, may counterbalance early life stressors, contributing to lower or commensurate levels of depression in Black youth and White youth (Kysar-Moon, 2020; Louie et al., 2022; Louie & Wheaton, 2019). However, newly emerging research, some in nationally representative samples, suggests that racial and ethnic minorities are now experiencing greater growth and higher rates in depressive symptoms during adolescence and early adulthood compared to White individuals (Daly, 2022; Hargrove et al., 2020; Lu, 2019). These findings highlight the importance of identifying etiological predictors and outcomes of growth in depressive symptoms across childhood and adolescence in diverse racial/ethnic subgroups.

### Depressive symptoms and substance use

Alcohol, cannabis, and tobacco use are common correlates of depressive symptoms in adolescence (Chadi et al., 2019; Maughan et al., 2013). However, not all studies find depressive symptoms associated with substance use (Assari et al., 2018). There is also evidence that childhood and adolescent depressive symptoms increase risk for substance use outcomes across sex and race (Copeland et al., 2021). These findings are corroborated by the few studies demonstrating that accelerated growth in depressive symptoms across childhood and adolescence is associated with higher rates of adolescent substance use (Edwards et al., 2014; Leve et al., 2012). Theoretical perspectives postulate that increases in depressive symptoms across adolescence are associated with higher stress reactivity, psychosocial stress, and social isolation (Chaplin et al., 2018; Trucco, 2020). In turn, it has been postulated that depressed youth use substances to self-medicate to relieve depressive symptoms and associated stresses (Bernaras et al., 2019; Hussong et al., 2017; Trucco, 2020). An alternative but complementary perspective is that shared etiological factors underlie both depression and substance use (Garey et al., 2020), an association that has previously been found among low-income Black and White emerging male adults (Womack et al., 2016). Conversely, substance use in adolescence often occurs with peers, and depressed youth may have fewer interactions with peers (Trucco, 2020), so it is possible that increased growth in depressive symptoms might be linked to lower rates of substance use. More recent research has linked growth in depressive symptoms across adolescence to use of e-cigarettes (Moustafa et al., 2021). However, research has yet to link growth in depression symptoms across childhood and adolescence to substance use in adolescence.

### Intervention effects on depressive symptoms and substance use

A meta-analysis and two reviews indicate that psychosocial interventions have small to moderate effects on adolescent depressive symptoms and substance use (Cuijpers et al., 2021; Das et al., 2016; Kuntsche & Kuntsche, 2016). One strategy these interventions utilize is supporting positive parenting practices (e.g., greater proactive involvement and monitoring of youth activities, improving parent-youth communication) to help prevent youth psychopathology. In the current study, families were randomized to the FCU or a control condition at child age two. This preventive intervention has shown effects on adolescent depression as part of integrative data analysis across three combined samples (Connell et al., 2021). In other samples that conducted trials of the FCU, intervention effects have been found on increasing adolescent depression and growth in marijuana use (Connell et al., 2018; Véronneau et al., 2016). Although not a primary aim of the current study, we examined for intervention effects on substance use based on prior evidence.

### Genetic predisposition, intervention, and depressive symptoms

There is converging evidence from twin and molecular genetic designs that there are genetic effects on depressive symptoms during childhood and adolescence. For instance, twin studies indicate that depressive symptoms in childhood are largely attributable to environmental factors and that moderate genetic influences emerge in early adolescence (Bergen et al., 2007; Rice, 2009). However, some studies do find high heritability in childhood and increasing environmental effects over time (Nivard et al., 2015). These studies often conclude that continuity in depression over time is primarily due to genetic effects, but unique genetic influences can exist during different developmental periods. Molecular genetic work finds that genetic variability peaks in adolescence (Sallis et al., 2017). A recent review of molecular genetic research illustrated that genetic candidate variants are associated with early adolescent depressive symptoms (Akingbuwa et al., 2022).

As an extension of this work, more recent research has examined polygenic scores (PGS) for depression which aggregate across heterogeneity in thousands of genetic variants across the genome. This approach is useful as it captures the underlying polygenic architecture of depression. A handful of studies have examined polygenic associations with childhood and adolescent depressive symptoms using the Avon Longitudinal Study of Parents and Children. For instance, Kwong et al. (2021) examined trajectories of depressive symptoms using multilevel growth curve modeling, finding PGS for broad depression and for major depressive disorder (MDD) associated with steeper trajectories and greater rate of change of depressive symptoms from early adolescence to early adulthood (Kwong et al., 2021). In another study using growth mixture modelling, trajectories characterized by high levels of depression were associated with a PGS for depressive symptoms from age 10 to 24 (Kwong et al., 2019). Similarly, a PGS for MDD was associated with late-onset depressive symptom trajectories across adolescence (Rice, 2009) and trajectories with elevated depressive symptoms from age 4 to 17 (Lussier et al., 2021).

Despite evidence of polygenic effects on trajectories of depressive symptoms, the majority of this research has been conducted in samples of White individuals, so it is unclear if findings would generalize to Black individuals. One important distinction is that race and ethnicity are social constructs rather than based on genetic ancestry (Feero et al., 2024; National Academies of Sciences, Engineering, and Medicine [NASEM], 2023). Polygenic research often uses genome-wide association studies (GWAS) conducted in White or European American populations to create polygenic scores for use with other races and ethnicities. This strategy is methodologically and conceptually inaccurate because genetic architecture may vary across race and ethnicity so polygenic scores are most predictive when summary statistics from the independent GWAS are from the same or similar genetic ancestry as the replication sample in which polygenic scores are examined (Peterson et al., 2019).

In one recent study addressing this concern, Elam et al. (2024) leveraged separate GWAS on depression in Black and White samples to create polygenic scores for depression for youth in the Adolescent Brain Cognitive Development (ABCD) study. Depression polygenic scores were associated with elevated trajectories of depressive symptoms from age 9/10 to 12/13 in White youth but not Black youth. Based on the young age of participants and low prevalence of substance use in ABCD, intent to use substances was examined as the outcome. Within White youth, depressive symptom trajectories were associated with greater intent to use substances. Conversely, high and persistent depressive symptom trajectories in Black youth were associated with less intent to use substances. These results illustrate the complex interplay among polygenic predisposition for depression, depressive symptoms, and substance use outcomes across race and highlight the importance of using racially aligned polygenic scores, which we address in the current study. Developmental theory and research support that genetic predisposition for psychopathology may be associated with substance use in adolescence via growth in depressive symptoms over time (Elam et al., 2023; Trucco, 2020). For example, genetic predisposition for depression may contribute to the emergence of depression over time which, in turn, increases risk for substance use in adolescence. Thus, growth in depression may mediate genetic effects on substance use.

In addition to genetic main effects, there is growing evidence that genetic effects can be moderated by intervention effects. Broader developmental theoretical perspectives have proposed that psychopathology emerges in adolescence based on the interplay among multilevel individual and social mechanisms (Cicchetti & Rogosch, 2002). Frameworks on genetic-environment interplay posit that psychosocial interventions can buffer the emergence of maladaptive behavior associated with genetic predisposition by supporting and reinforcing involvement in prosocial environments, which, in turn, can prevent developmental cascades leading to psychopathology (Elam et al., 2023). This theory is supported by a recent review on genetic moderation of family-focused interventions, finding evidence of gene-by-intervention (GxI) effects on parent outcomes including maternal depression (Li et al., 2022). In the current sample, past studies have found children at high genetic risk were more likely to be in a persistent high trajectory group of conduct disorder from age 2–14 if they were in the control group but the persistent low trajectory group if they received the FCU (Shaw et al., 2019). We also found polygenic scores for aggression were associated with peer rejection in middle childhood which subsequently predicted adolescent marijuana use, but the FCU buffered both associations (Elam et al., 2022). Finally, a polygenic score reflecting genetic susceptibility for emotional problems was moderated by the FCU effects on internalizing problems in which the FCU led to fewer symptoms for genetically susceptible youth (Lemery-Chalfant et al., 2018). In another sample which also conducted a trial of the FCU, the association between genetic risk for aggression and substance use disorder in adulthood was buffered by the FCU intervention (Elam et al., 2021). Thus, there is evidence that psychosocial interventions can buffer both the development of psychopathology and substance use.

We note that polygenic scores have lower predictive power and accuracy when the genetic ancestry of the discovery GWAS sample does not align with the ancestry of the target polygenic sample (Martin et al., 2017). The genetic architecture of complex traits, such as depression, can vary across populations because of differences in genetic allele frequencies, linkage disequilibrium patterns, and allele effect sizes (Kachuri et al., 2024; Peterson et al., 2019). Based on these findings, it is important to align the genetic ancestry of the discovery GWAS and target polygenic population as close as possible. For example, one best practice would be to leverage an African-ancestry GWAS to create polygenic scores in an African-ancestry target sample. Also, because of population differences in genetic architecture, polygenic effects can operate differently across ancestry groups, increasing the probability that polygenic effects would not be statistically comparable across populations (Martin et al., 2017). Thus, polygenic effects in an African-ancestry group should not be equated to polygenic effects in European-ancestry groups. Rather statistical models are typically computed within each ancestral group without cross-comparison (Peterson et al., 2019). In the current study, we follow these best practices by aligning genetic ancestry across our discovery GWAS and target polygenic samples and computing models separately within African-ancestry and European-ancestry groups without statistical comparison.

### Current study

In the current study, we examined growth in depressive symptoms from age 7.5 to 16 separately in European-ancestry and African-ancestry youth of the FCU. For reasons described above, we examined genetic effects separately within each racial/ethnic subgroup. We created polygenic scores for depression (Dep-PGS) based on GWAS of depression from Levey et al. (2021), who conducted separated GWAS on MDD in 250,215 European-ancestry individuals and 59,600 African-ancestry individuals from the Million Veterans Program. We examined e-cigarette, alcohol, and cannabis use substance outcomes at age 16. We chose to examine e-cigarette use as the prevalence was nearly twice that of conventional cigarette use, reflecting recent trends. We examined whether growth in depressive symptoms mediated the association between Dep-PGS and substance use. Finally, we examined whether the FCU intervention had a buffering effect (GxI) on genetic associations between growth in depression and adolescent substance use.

We hypothesized that we would find increasing growth in depressive symptoms across time in European-ancestry and African-ancestry youth. Furthermore, we hypothesized that growth in depressive symptoms would be associated with higher rates of substance use in European-ancestry and African-ancestry youth and that depressive symptom growth would mediate associations between genetic risk and substance use. Lastly, we hypothesized we would detect GxI effects on depressive symptom trajectories such that positive genetic effects would be observed in the control group and null genetics effects in the intervention group.

## Method

### Participants

Seven hundred and thirty–one ethnically and racially diverse, low-income families with 2–year-old children were recruited between 2002 and 2003 from Women, Infants, and Children Nutritional Supplement Programs (WIC) at three sites located in metropolitan Pittsburgh, Pennsylvania (urban), Eugene, Oregon (suburban), and outside Charlottesville, Virginia (rural). Screening procedures were used to recruit families of toddlers at high risk for conduct problems, based on the presence of at least two of the following risk factors: sociodemographic risk, primary caregiver risk, and toddler behavior problems. Participation rates of those families invited to participate who qualified by risk status were high across the three sites [83.2% total (49% female); 84% in Eugene (*n* = 271), 76% in Charlottesville (*n* = 188), and 88% in Pittsburgh (*n* = 272)]. More than two thirds of the families reported an annual income of less than $20,000, with 24% of primary caregivers having less than a high school education, 41% having a high school diploma or general education diploma, and an additional 32% having 1–2 years of more than high school education. Primary caregivers (96% mothers) self-identified as belonging to the following ethnic groups: 11% Hispanic, 28% Black/African American, 54% White, 4% biracial, and 3% other groups (e.g., Native American, Asian American, Pacific Islander). For more information about sample characteristics, see Dishion et al. (2008).

Families were randomly assigned to control or intervention conditions after the baseline assessment at child age 2 years. Those in the control condition received WIC services as usual. Those in the intervention condition had the opportunity to receive the FCU following each of eight assessments occurring nearly annually from ages 2 to 10.5. Incorporating motivational interviewing, the FCU includes a formal assessment of youth and family factors that have been found to predict youth problem behavior, then sharing with the family the revealed concerns and family strengths during a feedback session to both motivate change but also demarcate assets the family can build on to address youth risk for problem behavior. At the end of the feedback session, parents identify goals they have for their child and family in the coming year and are given the option to work with the family coach on the family issues using evidence-based family management practices. In clinical practice, an initial interview precedes the assessment component, but to ensure that control and intervention families had comparable levels of contact with research staff, the assessment was convened for families in both the control and intervention groups, but only intervention group families were offered the initial interview, feedback, and follow-up parent management sessions. All families were re-contacted at child ages 3, 4, 5, 7.5, 8.5, 9.5, 10.5, 14, and 16 years (81% of the sample participated at age 16). In terms of engagement in the intervention among intervention families, 76% of families engaged at age 2, with over 90% of the families engaging in at least one session of the FCU by child age 5.

For the current study, we utilized data from the age 7.5, 8.5, 9.5, 10.5, 14, and 16 assessments. Of the adolescents who participated at 14 years old, 515 were genotyped (86.7% of the sample who participated in home visits at age 14). The current study leveraged subsamples of adolescents who were genotyped and using ancestry principal components were identified as African-ancestry (*n* = 154; 50% Intervention group) or European-ancestry (*n* = 219; 51% Intervention group). Depressive symptom data were available for greater than 85% of the sample at all ages (age 7.5: *n* = 329, 87%; age 8.5: *n* = 316, 84%; age 9.5: *n* = 342, 91%; age 10.5: *n* = 335, 89%; age 14: *n* = 364, 97%; age 16: *n* = 355, 95%). Selective attrition analyses revealed no difference in child gender, race, or intervention group for those with vs. without data on depressive symptoms at age 16. Youth with missing depressive symptom data at age 16 did have higher depressive symptoms at age 7.5 and age 8.5 than those without missing data; however, the number missing was small (*n* = 13, *n* = 11). There were no significant differences between members of the initial sample who did not provide genetic data and those who were genotyped with respect to parental education, race, gender, study site, child problem behaviors at age 2, temperament, or parental depression.

### Procedures

All assessments were conducted in the home at ages 2 to 16 with primary caregivers (96% biological mothers at age 2) and children. Primary caregivers completed questionnaires regarding the physical and sociocultural context and children’s behavior. All study protocols were approved by the respective university Institutional Review Boards, parental written consent was obtained for all families (with assent obtained from children beginning at age 14), and families were compensated for their time at each age.

Participants provided saliva samples with Oragene kits for genotyping during the age 14 home visit. RUCDR Infinite Biologics at Rutgers University extracted and normalized the DNA and then genotyped the samples using the Affymetrix Axiom Biobank1 Array. SNPs that did not meet the criteria of Hardy–Weinberg equilibrium at *p* < 10^-6^ and SNPs with a minor allele frequency less than 1% were removed. Also, any SNP or individual with a missing data rate greater than or equal to 5% was removed (no participants met the Hardy–Weinberg criteria).

## Measures

### Polygenic risk scores for Depression (Dep-PGS)

We created Dep-PGS based on separate GWAS summary statistics from African-ancestry and European-ancestry subsamples. Levey et al. (2021) conducted separate GWAS in African-ancestry (*N* = 250,215) and European-ancestry (*N* = 59,600) individuals from the Million Veterans Program on MDD (Levey et al., 2021). We used separate summary statistics from the GWAS in African-ancestry and European-ancestry samples to form Dep-PGS using PRS-CSx in African-ancestry and European-ancestry youth in the current study, respectively. PRS-CSx uses Bayesian regression and continuous shrinkage to include single nucleotide polymorphisms (SNPs) across the genome (Ruan et al., 2022). PRS-CSx methods improve polygenic prediction and predictive power across multiple racial/ethnic populations beyond traditional methods of polygenic construction (Ge et al., 2019; Ruan et al., 2022). Summary statistics for African-ancestry and European-ancestry Dep-PGS were drawn from the respective GWAS with joint modelling across GWAS summary statistics using coupled shrinkage priors. Final Dep-PGS were generated separately for African-ancestry and European-ancestry participants based on posterior PRS-CSx weights and using the *score* procedure in PLINK 1.9 (Chang et al., 2015). An overview on polygenic score methods in diverse populations can be found in Kachuri et al., (2024).

### Population genetic admixture

We conducted a principal components analysis of all autosomal SNPs to represent population admixture using PLINK (see Price, Patterson, Plenge, Weiblatt, Shadick, & Reich, 2006) (Price et al., 2006). Prior to extracting principal components, we screened out regions of long-range linkage disequilibrium (LD; correlation among the SNPs) and used PLINK’s sliding window procedure to prune local LD. We extracted the first 20 components. The first component (PC1) had an eigenvalue of 28.84 and differentiated European-ancestry and Latino groups from African-ancestry groups. The second component (PC2) had an eigenvalue of 5.62 and differentiated non-Latino participants (European-ancestry and African-ancestry) from Latino participants. Principal components reflecting population structure based on genome-wide data were used to assign European-ancestry (*n* = 219) and African-ancestry (*n* = 154) (Peterson et al., 2019). Also, the first 10 principal components were regressed from polygenic scores and the unstandardized residuals were saved, which were subsequently standardized for ease of interpretation.

### Child/adolescent depressive symptoms

Primary caregivers completed the Child Behavior Checklist 6–18 (Achenbach et al., 2001) at the age 7.5, 8.5, 9.5, 10.5, 14, and 16 assessments. Parents rated each item on a 3-point scale (0 = not true, 1 = somewhat or sometimes true, 2 = very true or often true). The depressed/withdrawn subscale was used in the current analyses, which assesses children’s depressive behaviors (e.g., “Unhappy, sad, or depressed”). Internal consistency was good at all assessments (αs range from .72 to .84).

### Youth substance use

At age 16, target adolescent participants completed 23 items assessing use of tobacco/nicotine, alcohol, cannabis, illicit drugs, and prescription substances (Dishion et al., 2008). For the present study, we utilized separate items reflecting the frequency of alcohol, cannabis, and e-cigarette use over the past 3 months, which were selected as they had the highest prevalence (0 = never, 1 = once or twice, 2 = once a month, 3 = once every 2–3 weeks, 4 = once a week, 5 = 2–3 times a week, 6 = once a day, 7 = 2–3 times a day or more). The three items were used as indicators for a latent variable reflecting substance use frequency at age 16. The unconstrained latent model was a good fit to the data (RMSEA = 0.0, CFI/TLI = 1.0) with significant loadings (alcohol frequency: .90, e-cigarette frequency: .42; marijuana frequency: .61).

### Covariates

In all models we covaried for child gender and location (rural, urban, suburban). In addition, the first 10 principal components were residualized from the Dep-PGS to account for potential population stratification. Following field recommendations (Keller, 2014), we also examined two-way polygenic by covariate interactions and retained interaction terms when significant.

## Analytic approach

Within the analytic sample, there were missing data across waves of depressive symptoms and for indices of substance use for European-ancestry participants (depressive symptoms: 1% to 14%; substance use: 5% to 6%) and African-ancestry participants (depressive symptoms: 4% to 18%; substance use: 6% to 7%). Missing data were handled using Full Information Maximum Likelihood (FIML). Outliers on Dep-PGS and phenotypic variables were trimmed.

Mean levels and significant differences among phenotypic study variables across racial/ethnic groups were examined using MANOVA in SPSS v28. Following these steps, we examined growth in depressive symptoms at ages 7.5, 8.5, 9.5, 10.5, 14, and 16 using latent growth curve models (LGCM) in Mplus v.8.8 separately for African-ancestry and European-ancestry subgroups. We took this approach to examine genetic effects separately in each subgroup based on potential differences in the Dep-PGS functioning due to possible underlying variation across subgroups in genetic ancestry (e.g., allele frequency, linkage disequilibrium patterns) (Feero et al., 2024; Martin et al., 2017; NASEM, 2023).

We first examined unconstrained LGCM models including intercept and slope terms to examine mean and variance in intercept and slope terms. Following this step, within LGCM we examined Dep-PGS, intervention group, and covariates as predictors of intercept and slope. Within the same model we examined the substance use latent variable (e-cigarette, alcohol, and cannabis use frequency) as an outcome of the intercept and slope, and also as outcomes of the Dep-PGS, intervention group, and covariates. Finally, in a subsequent model we included the Dep-PGS by intervention group interaction term as a predictor of intercept, slope, and the substance use latent variable. We present standardized effect sizes for all models in the text and tables. To note, methodological studies on power in latent growth curve models (LGCM) indicate a sample size of 100–150 individuals is recommended for a LGCM containing a quadratic term with 4 waves of measurement (Diallo et al., 2014). In our model we had six waves of measurement and a minimum sample size of 154 in the smaller African-ancestry group indicating our LGCM was adequately powered. In addition, other studies suggest that the ability to reject the null in LGCM is based on adequate value of RMSEA (Preacher, 2018).

Where significant interaction effects were observed, we examined the Dep-PGS effects separately in the control and intervention groups using multigroup modeling. Where significant associations were detected on intercept and slope and also from intercept and slope to substance use, we tested for mediation using the R package “Rmediation” (Tofighi & MacKinnon, 2011). As sensitivity tests, we examined LGCM for a subset of ages in which we observed change in depressive symptoms to examine for specificity in associations with predictors and outcomes.

## Results

**Figure 1.**
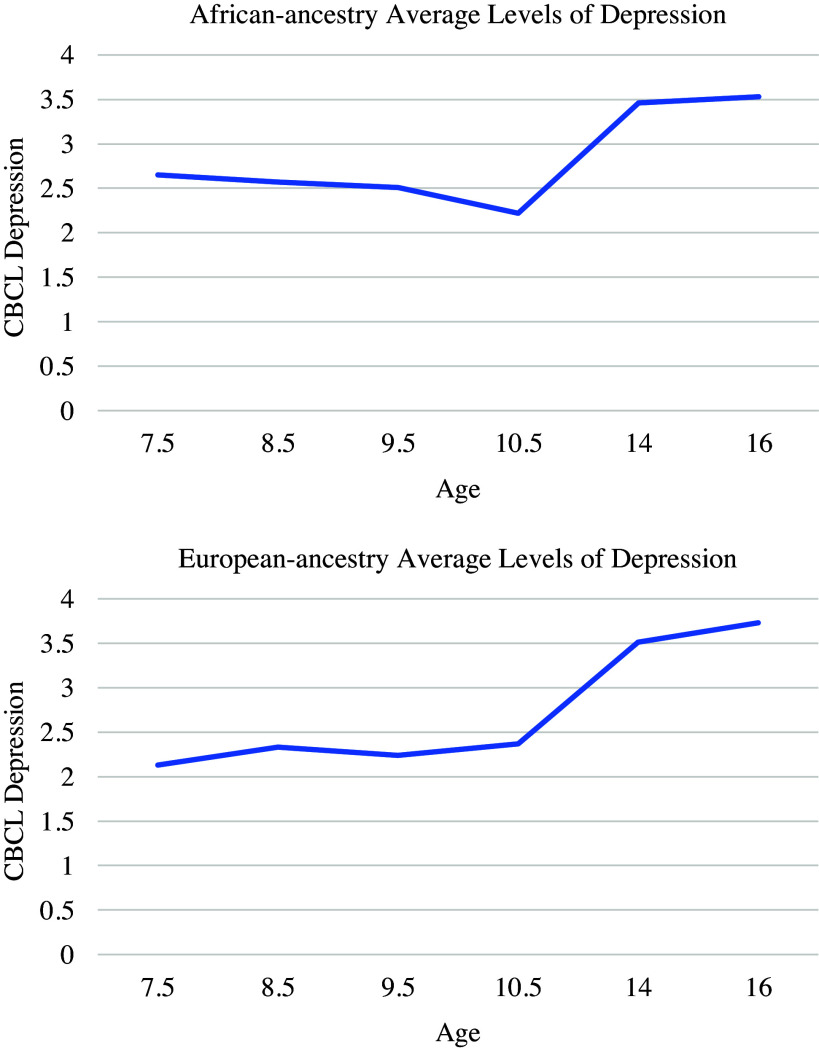
African-ancestry and European-ancestry average depression from age 7.5 to 16.

Descriptive statistics can be found in Table 1. African-ancestry and European-ancestry youth did not vary on levels of depressive symptoms at any age. Levels of substance use were relatively low in both groups, with European-ancestry youth having greater alcohol and e-cigarette use compared to African-ancestry youth at age 16. Average levels of depressive symptoms for both groups remained relatively stable from age 7.5 to 10.5 after which they increased sharply to age 14 with slight upward trends to age 16 (Figure 1).

**Table 1.** Descriptive statistics for study variables and differences tests

	European-ancestry Youth Mean (SD)	African-ancestry Youth Mean (SD)	*F (df), P value*
Depressive symptoms 7.5	2.13 (2.35)	2.65 (2.50)	3.70 (1, 328), *p* = .055
Depressive symptoms age 8.5	2.33 (2.42)	2.57 (2.85)	2.49 (1, 315), *p* = .116
Depressive symptoms age 9.5	2.24 (2.20)	2.51 (2.81)	.97 (1, 341), *p* = .327
Depressive symptoms age 10.5	2.37 (2.61)	2.22 (2.46)	.27 (1, 334), *p* = .607
Depressive symptoms age 14	3.51 (3.45)	3.46 (3.40)	.02 (1, 363), *p* = .904
Depressive symptoms age 16	3.73 (3.29)	3.53 (3.47)	.31 (1, 354), *p* =.577
E-Cigarette Frequency	**.52 (1.42)**	**.18 (.79)**	**6.83 (1, 348), *p* = .009**
Alcohol Frequency	**.52 (1.07)**	**.26 (.84)**	**5.96 (1, 349), *p* = .015**
Cannabis Frequency	.71 (1.70)	.62 (1.67)	.25 (1, 348), *p* = .619
Gender (% Female)	48%	50%	.15 (1, 372), *p* = .697

*Note.* Significant difference across European-ancestry vs. African-ancestry at *p* < .05 are bolded.

We first examined unconstrained LGCM across ages 7.5 to 16 separately in African-ancestry and European-ancestry youth. The unconstrained model in African-ancestry youth had a good fit (*X* ^*2*^ = 34.45, *p* = .003, RMSEA = .09, CFI = .96, TLF = .96) and significant estimates for the mean and variance of the intercept and slope (mean intercept = 2.47, slope = .12; variance intercept = 4.91, slope = .08, *ps* < .001), and the intercept and slope were negatively correlated (*r* = −.29, *p* = .011). The unconstrained model in European-ancestry youth had a good fit (*X* ^*2*^ = 33.52, *p* = .006, RMSEA = .072, CFI = .96, TLF = .96) and significant estimates for the mean and variance of the intercept and slope (mean intercept = 1.90, slope = .21; variance intercept = 3.06, slope = .05, *ps* < .001), but the intercept and slope were not significantly correlated (*r* = −.14, *p* = .26).

We next examined main effects among the Dep-PGS, depression intercept and slope, and the substance use latent factor (see Table 2). Within the African-ancestry group, the Dep-PGS was negatively associated with the substance use latent factor. The slope of the depression trajectory was positively associated with the substance use latent factor. Within the European-ancestry group, the Dep-PGS was negatively associated with the slope of depression.

**Table 2.** Main effects on latent growth curve model for African-ancestry and European-ancestry youth

	Depression Trajectory Age 7–16			Depression Trajectory Age 10.5–16		
	I	S	SU Latent	I	S	SU Latent
**African-ancestry Youth**	b (SE), *p* - value	b (SE), *p* - value	b (SE), *p* - value	b (SE), *p* - value	b (SE), *p* - value	b (SE), *p* - value
Dep-PGS	−0.03 (.09), .744	0.03 (.11), .820	**-0.16 (.07), .015**	.09 (.09), .308	−.05 (.11), .640	**−.16 (.07), .023**
Group	−0.05 (.09), .599	0.04 (.11), .737	0.10 (.07), .156	.00 (.09), .959	−.02 (.11), .835	.13 (.07), .088
Gender	−0.01 (.09), .896	0.05 (.11), .628	0.02 (.06), .735	.01 (.09), .950	.03 (.10), .755	.03 (.07), .675
Location	0.13 (.09), .141	−0.16 (.11), .137	0.06 (.07), .333	.07 (.09), .406	−.14 (.11), .216	.04 (.07), .564
I	–	–	−0.11 (.07), .120	–	–	−.03 (.09), .789
S	**−.30 (.11), .007**	–	**0.20 (.09), .028**	−.23 (.29), .426	–	.16 (.11), .156
**European-ancestry Youth**						
Dep-PGS	−.04 (.08), .633	**-.24 (.10), .020**	−.14 (.09), .092	−.11 (.07), .100	−.11 (.08), .176	**−.17 (.08), .040**
Group	−.06 (.08), .449	.16 (.10), .131	−.09 (.09), .318	.03 (.07), .609	.07 (.08), .408	−.06 (.08), .452
Gender	−.02 (.08), .804	.01 (.11), .909	.07 (.08), .408	−.10 (.07), .126	.13 (.08), .105	.07 (.09), .398
Location	.04 (.08), .671	−.12 (.11), .272	.15 (.09), .072	−.00 (.07), .972	−.06 (.08), .497	.13 (.08), .114
I	–	–	−.05 (.10), .587	–	–	.07 (.12), .593
S	−.15 (.13), .242	–	.20 (.14), .145	**−.55 (.12), .000**	–	.12 (.13), .350

*Note*. *I* = intercept; *S* = slope; SU = substance use; Dep = Depression; PGS = polygenic score. The association between the intercept (I) and slope (S) terms reflects the Intercept-Slope correlation within the latent growth curve model. Bolded estimates are significant at *p* < .05.

**Figure 2.**
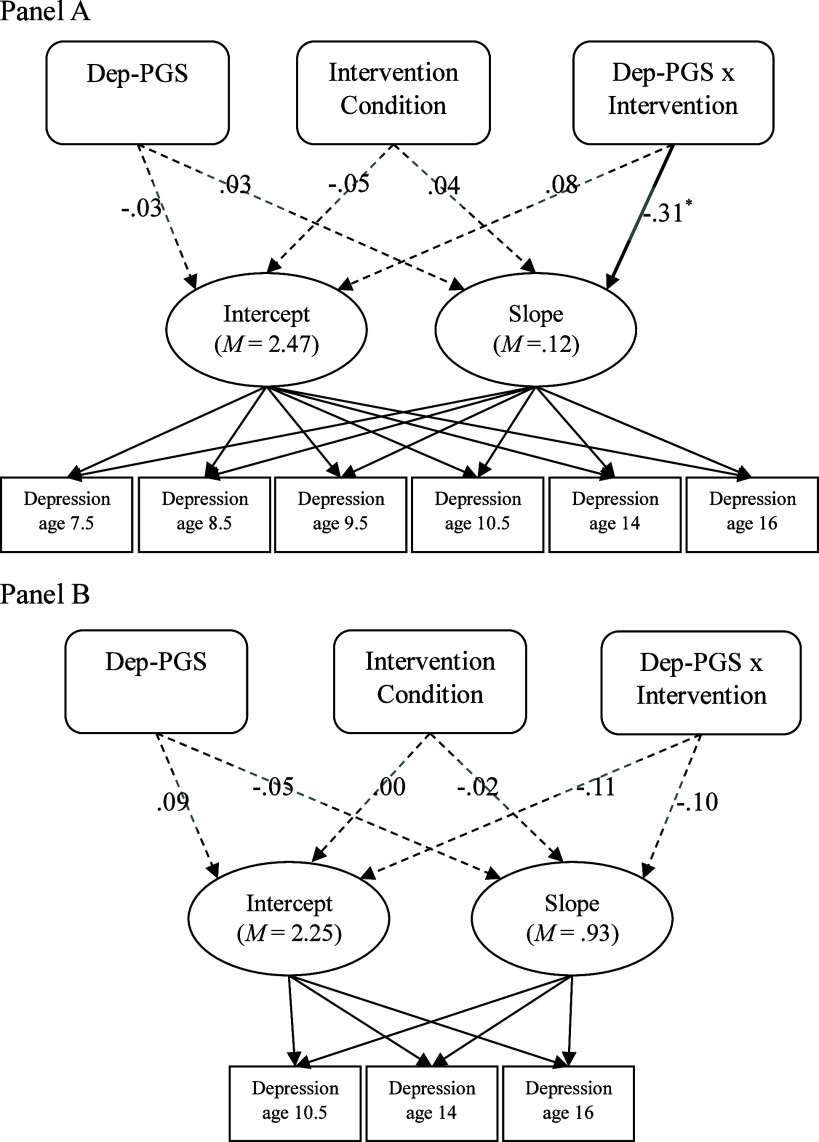
African-ancestry latent growth curve results for the 7.5- to 16-year-old model (panel A) and 10.5- to 16-year-old model (panel B). *Note.* Significant path estimates are bolded at *p* < .05 and represented by solid lines. Nonsignificant paths are represented by dashed lines.

**Figure 3.**
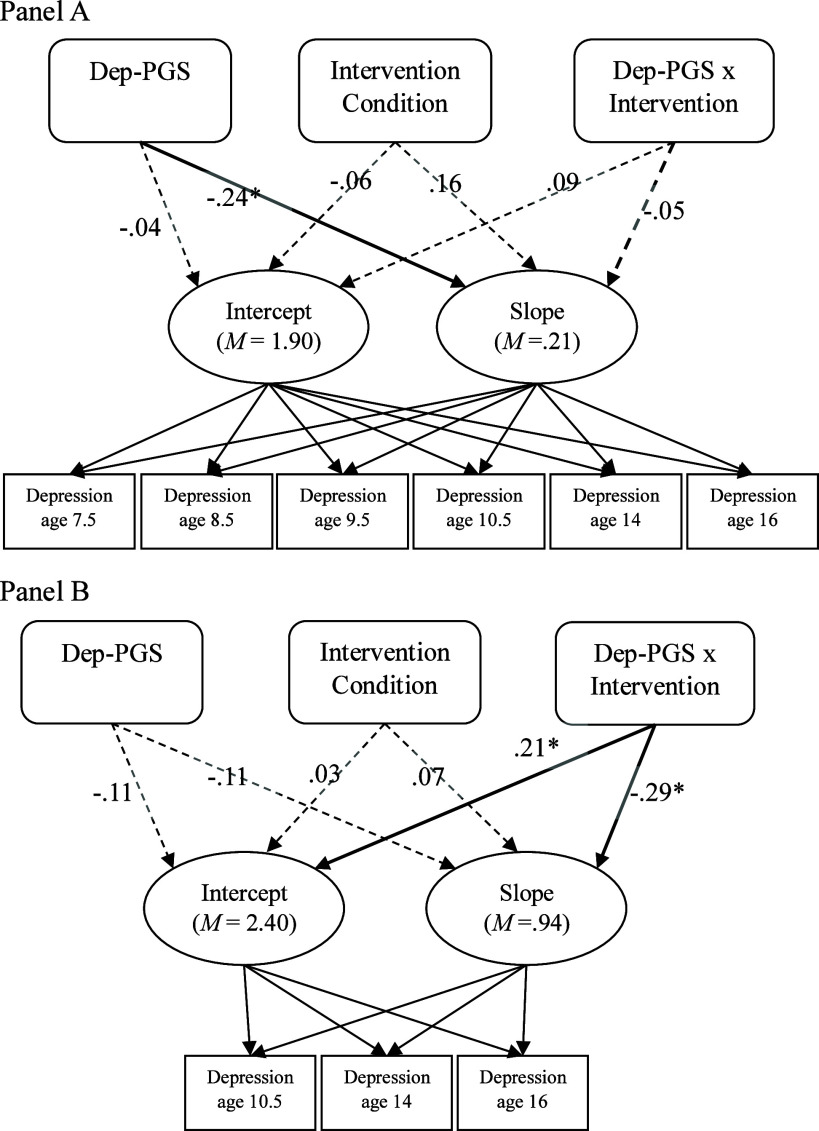
European-ancestry latent growth curve results for the 7.5- to 16-year-old model (panel A) and 10.5- to 16-year-old model (panel B). *Note.* significant path estimates are bolded at *p* < .05 and represented by solid lines. Nonsignificant paths are represented by dashed lines.

Next, we examined the Dep-PGS by intervention group interaction as a predictor of depression intercept, slope, and the substance use latent factor, in a model accounting for Dep-PGS, intervention group, and covariates. Model estimates can be found in Table 3 and Figures 2 and 3. Within the European-ancestry group, no GxI effects were observed on the intercept, slope, or the substance use latent factor. For African-ancestry youth, the Dep-PGS by intervention group interaction was negatively associated with the depression slope. To probe this interaction, we examined the association between the Dep-PGS and slope separately within the control and intervention groups. We found a significant positive association in the control group (*b* = .40, *p* = .029) and a nonsignificant negative effect in the intervention group (*b* = −.16, *p* = .242), which were significantly different (*X* ^*2*^ difference (1) = 5.31, *p* = .021). We also found the depression slope was associated with the substance use latent factor. There was a significant mediation effect from the Dep-PGS by group interaction term to the substance use latent factor via the slope in the overall model (*b* = −.07, SE = .05; 95% CI: −.18, −.001).

**Table 3.** Interactive effects on latent growth curve model for African-ancestry and European-ancestry youth

	Depression Trajectory Age 7–16			Depression Trajectory Age 10.5–16		
	I	S	SU Latent	I	S	SU Latent
**African-ancestry Youth**	b (SE), *p*-value	b (SE), *p*-value	b (SE), *p*-value	b (SE), *p*-value	b (SE), *p*-value	b (SE), *p*-value
Dep-PGS by Group	.08 (.13), .545	**−.31 (.15), .048**	−.08 (.12), .515	−.11 (.13), .427	−.10 (.16), .543	−.14 (.09), .054
**European-ancestry Youth**						
Dep-PGS by Group	.09 (.12), .462	−.05 (.16), .772	.03 (.12), .827	**.21 (.10), .048**	**−.29 (.13), .020**	.03 (.12), .786

*Note.* I = intercept; S = slope; SU = substance use; Dep = depression; PGS = polygenic score. Bolded estimates are significant at *p* < .05.

Within the European-ancestry group, no GxI effects were observed on the intercept, slope, or the substance use latent factor. However, we examined the association between the Dep-PGS and slope separately in the control and intervention group to probe the negative Dep-PGS main effect on the depression slope. There was a significant negative association in the intervention group (*b* = −.37, *p* = .032) and a nonsignificant negative effect in the control group (*b* = −.12 *p* = .357), however, these effects were not significantly different (*X* ^*2*^ difference (1) = .47, *p* = .494).

As part of sensitivity analyses, we observed the greatest change in mean levels of depression over time in African-ancestry and European-ancestry youth from age 10.5 to 16 (see Table 1). We re-examined a second set of LGCM for African-ancestry and European-ancestry youth from ages 10.5 to 16, again examining predictors and outcomes specific to change in depressive symptoms and the substance use latent factor. Model results can be found in Table 2 and 3. For African-ancestry youth, there were no significant main or interactive effects on the intercept or slope of depression, but the Dep-PGS was negatively associated with the substance use latent factor.

In European-ancestry youth, the Dep-PGS was negatively associated with the substance use latent. When examining interactive effects, the Dep-PGS by intervention group interaction was positively associated with the depression intercept and negatively associated with the depression slope. When probing the interaction on the intercept, we found a negative association between the Dep-PGS and intercept in the control group (*b* = −.30, *p* = .017) and a nonsignificant positive association in the intervention group (*b* = .01, *p* = .884), which were significantly different (*X* ^*2*^ difference (1) = 4.63, *p* = .031). When probing the interaction on the slope, we found a positive but nonsignificant association between the Dep-PGS and slope in the control group (*b* = .21, *p* = .245) and a significant negative association in the intervention group (*b* = −.24, *p* = .027), which were significantly different (*X* ^*2*^ difference (1) = 7.13, *p* = .008).

## Discussion

Despite a robust literature examining growth in depressive symptoms across childhood and adolescence, relatively few studies have examined genetic predictors, and even fewer have included substance use outcomes or used diverse samples. Leveraging a diverse sample, we examined whether polygenic predisposition for depression predicted growth in depression symptoms from age 7.5 to 16, trajectory associations with substance use, and moderation of genetic effects by a family-centered intervention. We note that we conducted analyses separately for African-ancestry and European-ancestry subgroups and do not make comparisons or interpret differences across groups because polygenic effects can vary across ancestral populations due to possibly underlying variation in genetic ancestry (Feero et al., 2024; Martin et al., 2017; NASEM, 2023) and cultural practices. We did detect a negative main genetic effect on growth in depressive symptoms in European-ancestry youth. There was also evidence of moderation of genetic effects by the FCU intervention on growth in depressive symptoms in both the African-ancestry and European-ancestry groups, and growth in depressive symptoms was associated with greater substance use in African-ancestry youth. These findings highlight the utility of early family-based interventions in buffering long-term genetic effects on youth mental health.

Consistent with hypotheses, we found increasing growth in depressive symptoms in both African-ancestry and European-ancestry youth, particularly from age 10.5 to 16. This finding aligns with past research demonstrating normative increases in depressive symptoms during the transition from childhood to adolescence (Musliner et al., 2016; Shore et al., 2018). We also hypothesized that polygenic prediction for depression would be associated with growth in depressive symptoms, which would in turn be associated with substance use as reflected by a mediated effect. Relatedly, we hypothesized GxI effects on depressive symptom trajectories such that positive genetic effects would be observed in the control group and null genetic effects in the intervention group. We only found partial support for these hypotheses.

In the age 7.5 to 16 trajectory model for African-ancestry youth, we found a GxI effect on the depression slope, and the slope was associated with the substance use latent factor. The slope in depressive symptoms mediated the relation between the Dep-PGS by intervention group interaction term and the substance use latent factor. We interpret this finding to indicate that greater polygenic predisposition for depression contributes to more frequent substance use in adolescence via steeper growth in depressive symptoms. However, when examining effects in the control and intervention subgroups, the Dep-PGS was associated with the slope only in the control group, and neither intercept nor slope was associated with substance use. This finding is likely due to a decrement in power due to the small sample size in the African-ancestry youth intervention (*n* = 77) and control (*n* = 77) groups. It should also be noted that the GWAS on African-ancestry individuals *(N* = 59,600) was relatively smaller than the GWAS on European-ancestry individuals *(N* = 250,125). In part, detection of polygenic effects and polygenic effect size are based on the size of the GWAS discovery sample (Wray et al., 2014). Thus, smaller effect sizes or lack of effects in our African-ancestry sample may be due to the relatively smaller GWAS sample in Levey et al. (2021). In the age 7.5 to 16 trajectory model within the European-ancestry group the only effect we detected was a negative association between the Dep-PGS and the depression slope. When probing in the intervention vs. control groups we found a significant negative association in the intervention group and nonsignificant effect in the control group, although these were not significantly different. This finding may indicate a trend-level GxI effect that is underpowered as evidenced by the nonsignificant GxI interaction term.

Within the age 10.5 to 16 trajectory model in European-ancestry youth, we found a GxI effect on the slope in the age 10.5 to 16 trajectory model, such that in the control group there was a nonsignificant (but positive) effect and a significant negative effect in the intervention group. The negative genetic effect in the intervention group is somewhat counterintuitive but may indicate there are unmeasured ancillary effects of the intervention. For example, it could be that parents in the intervention condition are primed to notice youth behavioral symptoms, such as depression (reflective of youth’s genetic predisposition for depression), and proactively enact strategies provided by the FCU over time. Thus, youth with greater genetic predisposition for depression in the intervention condition may have less growth in depressive symptoms over time.

These findings add to a growing body of literature indicating that psychosocial interventions can buffer genetic predisposition for youth psychopathology and substance use outcomes (Elam et al., 2022; Neale et al., 2021). For example, in the current sample the FCU buffered the effects of polygenic predisposition for aggression on peer rejection in middle childhood and adolescent marijuana use (Elam et al., 2022). Also, the FCU led to fewer internalizing symptoms for genetically susceptible youth (Lemery-Chalfant et al., 2018). In a separate sample conducting a trial of the FCU, the association between genetic risk for aggression and substance use disorder in adulthood was buffered by the FCU intervention (Elam et al., 2021).

Of note, we did not find any other genetic main effects of the Dep-PGS on intercept or slope of depressive symptoms, or effects of intercept and slope on the substance use latent factor. The absence of these effects may be for several reasons. As demonstrated by some literature (e.g., Elam et al., 2024), it may be that there is heterogeneity in growth in depressive symptoms which we were unable to capture given our sample size. Relatedly, it may be that the sample size in our African-ancestry and European-ancestry groups was too small to detect genetic associations, especially in the context of a randomized intervention. Also, there are likely age-related differences in genetic effects which may have obscured findings. Examining polygenic effects in youth based off GWAS in older samples is common because of the dearth of GWAS in younger samples. Our Dep-PGS were based on a GWAS sample in middle to older adulthood. This method may have led to smaller effect sizes in our Dep-PGS. The magnitude of genetic effects on depression are known to vary across the lifespan, such that they are smaller in childhood but become larger into adolescence (Bergen et al., 2007; Rice, 2009). Thus, our study may have better captured genetic effects with more measurements of depressive symptoms in adolescence. Collectively, future research should endeavor to conduct GWAS in younger and more diverse populations and test polygenic effects across childhood and adolescence, helping to clarify the role of genetic effects on the development of psychopathology (Elam et al., 2023).

The lack of associations between growth in depressive symptoms and substance use is also surprising but may be indicative of a relative lack of frequent substance use in adolescence, a finding which is consistent with reductions in national trends (Ball et al., 2023). Collectively, it is likely that there are unmeasured social, contextual, and cultural risk and protective factors that contribute to change in depression and associations with substance use in adolescence (Patil et al., 2018). For instance, there is a robust literature indicating that harsh, rejecting parenting, experiences of discrimination, and neighborhood disadvantage contribute to adolescent depression (e.g., Cao et al., 2021; English et al., 2014; King et al., 2022). However, there are also commensurate protective factors such as supportive parenting and racial/ethnic identity which can buffer the association between disadvantage and negative experiences in depression (Cao et al., 2021; Rodriguez et al., 2009). These social, cultural, and contextual influences were not captured in our study but may have weakened genetic and phenotypic associations based on their robust association with adolescent depression.

The current study had several strengths, including examination of genetic effects in a diverse sample using racially aligned polygenic scores for depression. We were also able to examine for moderation of genetic effects by the FCU on longitudinal patterns of depression given multiple measurements waves. There were also limitations, including the need to examine effects separately by race, which resulted in a relatively small African-ancestry group. However, this approach is important in that it enables detection of specific effects for diverse youth and is necessitated by different patterns of linkage disequilibrium and allelic frequency in different races and ethnicities. Future research is needed in other racial/ethnic groups to help ensure equitable benefit of scientific discovery. Also, we leveraged parent report of depressive symptoms, in part, because youth reports were unavailable in childhood. While use of youth reports would have been preferable to reliance on parent reports during adolescence, research shows that parent reports of children’s depressive symptoms are fairly robust (Kim et al., 2016; Rey et al., 1992), and the findings linking parent reports of growth in depressive symptoms to youth reports of substance use cannot be attributed to reporter bias (i.e., using the same informant to report on both depression and substance use). Finally, the current sample included families with toddlers at high risk for conduct problems at recruitment. Therefore, results may not generalize to other populations.

The current findings have important implications. Specifically, growth in African-ancestry youth depressive symptoms was associated with substance use frequency at age 16. However, genetic associations on growth in depressive symptoms were buffered by the FCU. Thus, at-risk Black/African American youth may benefit from additional family-based supports early in life in buffering against depressive symptoms and substance use in adolescence.

## Data Availability

Behavioral data for ages 7–10 from this study are available from the Child & Family Data Archive (ICPSR 387407, 38745, 38748–49, 38751–55) by following data access procedures. Genetic data are available from dbGaP (phs003442.v1.p1) by following data access procedures. Other study data are available upon reasonable request from the principal investigators.
